# Influence of the Silver Nanoparticles (AgNPs) Formation Conditions onto Titanium Dioxide (TiO_2_) Nanotubes Based Electrodes on Their Impedimetric Response

**DOI:** 10.3390/nano9081072

**Published:** 2019-07-25

**Authors:** Marta Nycz, Katarzyna Arkusz, Dorota Genowefa Pijanowska

**Affiliations:** 1 Department of Biomedical Engineering, Faculty of Mechanical Engineering, University of Zielona Góra, Prof. Z. Szafrana 4, 54-516 Zielona Góra, Poland; 2 Nałęcz Institute of Biocybernetics and Biomedical Engineering, Polish Academy of Sciences, Ks. Trojdena 4, 02-109 Warszawa, Poland

**Keywords:** titanium dioxide (TiO_2_), titanium nanotubes (TNT), silver nanoparticles (AgNPs), electrochemical impedance spectroscopy, sputter deposition, chronoamperometry, cyclic voltammetry

## Abstract

This paper presents the comparison of the effects of three methods of production of silver spherical and near-spherical nanoparticles (AgNPs) on the titanium dioxide nanotubes (TNT) base: cyclic voltammetry, chronoamperometry, and sputter deposition. It also evaluates the influence of silver nanoparticles on the electrochemical properties of the developed electrodes. The novelty of this research was to fabricate regular AgNPs free of agglomerates uniformly distributed onto the TNT layer, which has not been accomplished with previous attempts. The applied methods do not require stabilizing and reducing reagents. The extensive electrochemical characteristic of AgNP/TNT was performed by open circuit potential and electrochemical impedance spectroscopy methods. For AgNPs/TNT obtained by each method, the impedance module of these electrodes was up to 50% lower when compared to TNT, which means that AgNPs enabled more efficient electron transfer due to the effective area increase. In addition, the presence of nanoparticles increases the corrosion resistance of the prepared electrodes. These substrates can be used as electrochemical sensors due to their high electrical conductivity, and also as implants due to the antibacterial properties of both the TNT and AgNPs.

## 1. Introduction

In recent years, the rapid development of sensors for biomedical applications has been observed because of the urgent need for non-standard diagnostic methods for fast and effective diagnosis of various disease entities. The sensor platform may be composed of many materials, the most frequently used being carbon [1,2,3] or silica-based compounds [4], polymers [5,6], or gold [7]. Platforms for sensors in the form of composites with two or more compounds [8] are becoming more and more popular. The main aim of using this kind of platform is to increase the sensitivity. Metal oxides and metallic nanostructures [9,10,11] are most widely applied. Among them, particular attention is paid to titanium dioxide nanotubes (TNT), which are characterized by high surface area, biocompatibility, good electrical conductivity, good adsorption properties, thermal and chemical stability, ease, and low production costs [12]. Improvement of the sensor sensitivity is obtained by the doping of metal nanoparticles, mainly gold nanoparticles (AuNPs) [13]. Silver nanoparticles (AgNPs) seem to be an alternative to these compounds. Its advantages are: ease and low cost of production, ease of binding with proteins in reaction with thiol group (–SH), and the best electrical conductivity among metals, so AgNPs may facilitate more efficient electron transfer than AuNPS. These properties make the AgNPs the most promising compound to be applied in electrochemical sensors [14,15,16,17]. However, the application of the AgNPs/TNT platform in sensing is rarely described [18,19]. At present, to the best of the authors’ knowledge, there are no publications reporting results on electrochemical characterization of the electrodes containing silver nanoparticles on titanium dioxide nanotubes. Most frequently, the characteristics of these platforms is limited only to evaluation of its photocatalytic and antimicrobial properties [20,21,22,23].

Besides traditional methods of silver nanoparticles production like chemical reduction of silver salts [24,25] using various (often expensive and toxic) reagents, new methods have been developed in recent years. The AgNPs have been produced by electrochemical methods [26], laser synthesis [27], sputter deposition [28], microwave irradiation [29], sonochemical synthesis [30] and photoreduction [31]. The methods of depositing AgNPs on TNT, which do not require the use of additional agents, are electrodeposition [32], i.e. potentiostatic [33,34] and galvanostatic [34] polarization, pulse method [35]. Similar results are obtained using sputtering [36]. However, the results obtained in previous studies [32,33] are not satisfactory due to the large number of agglomerates in the prepared substrates, or do not contain electrochemical analyses of these substrates [34,35,36].

The aim of this study was to compare the effects of three methods of silver spherical and near-spherical nanoparticles production on the titanium dioxide nanotubes base: cyclic voltammetry, chronoamperometry and sputtering, as well as to assess the influence of silver nanoparticles on the electrochemical properties of the developed electrodes. In the case of cyclic voltammetry, the variable parameter was the number of cycles, in the case of chronoamperometry and sputtering deposition time, so the influence of these parameters on the formation of agglomerates and the electrochemical characteristics of the samples were examined. For reference, an electrochemical study of TNT and TNT with a micro silver layer was also performed.

## 2. Materials and Methods 

### 2.1. Materials

Titanium foil (purity 99.7%), ethylene glycol (assay 99.8%), ammonium fluoride (purity ≥98%), phosphate buffered saline (0.01 M PBS, 0.0027 M potassium chloride and 0.137 M sodium chloride pH 7.4) were purchased from Sigma-Aldrich (St. Louis, MO, USA). Silver nitrate (AgNO_3_, analytical grade) was purchased from Stanlab (Lublin, Poland). All solutions were prepared from Milli-Q water.

### 2.2. TiO_2_ Nanotubes Fabrication

The titanium foil was sonicated in acetone, distilled water and dried under nitrogen atmosphere. The TiO_2_ nanotube layers were prepared by electrochemical anodization of titanium foils using Autolab PGSTAT302N (Metrohm, Herisau, Switzerland) at 17 V for 3750 s. Ethylene glycol (85% wt.) with ammonium fluoride (0.65% wt.) were used as the anodizing electrolyte. Scanning electron microscopy (FESEM, JEOL JSM-7600F, Tokyo, Japan) and energy-dispersive X-ray spectroscopy (EDS, INCA, Oxford Instruments, Oxford, UK) were used to investigate the surface morphology and chemical composition.

### 2.3. Thermal Modification

TNT layers were annealed in the AMP furnace (AMP, Zielona Góra, PL) in argon atmosphere at 450 °C for 2 h with the heating and cooling rate of 6 °C min^−1^. Annealing was performed to convert the as-formed (amorphous) structure of the nanotubes into the crystalline structure of anatase and rutile, which results in an increase in the conductivity of the TNT.

### 2.4. Modification of TNT with Silver Nanoparticles

#### 2.4.1. Cyclic Voltammetry Method (CV)

Deposition of AgNPs on TNT was carried out in 1 mM solution of AgNO_3_ in the potential range of −1.25 to −0.7 V in the three-electrode configuration with titanium dioxide nanotubular layer on the titanium foil as the working electrode, silver chloride electrode (E_Ag/AgCl_ = 0.222 V vs. Standard Hydrogen Electrode, SHE) by Metrohm as the reference electrode, and platinum mesh as the auxiliary electrode, with the scan rate of 0.05 V s^−1^ and a number of cycles in the range of 5–30. After deposition, the surface of the working electrode was washed with distilled water and dried under a nitrogen atmosphere. The samples will be hereinafter called *x* cycles_AgNPs/TNT, where x stands for the number of cycles.

In addition, to investigate the effect of silver nanoparticles on electrochemical characterization of TNT layers, TNT with a micro silver layer, hereinafter referred to as Ag/TNT, was used to compare their performance. For this purpose, the micro silver layer was produced by cyclic voltammetry in 50 mM solution of AgNO_3_ in the potential range −1.25 to −0.7 V with the scan rate of 0.05 V s^−1^ for 25 cycles.

#### 2.4.2. Chronoamperometry Method (CA)

Deposition of AgNPs on TNT was carried out in 1 mM solution of AgNO_3_ with constant potential of −1.2 V in the three-electrode system described in 2.4.1., during 60–300 s. After deposition, the surface of the working electrode was washed with distilled water and dried under nitrogen atmosphere. The samples are hereinafter referred to as *x* chrono_AgNPs/TNT, where x stands for the time of deposition.

#### 2.4.3. Sputter Deposition (SD)

For the deposition of AgNPs a sputter coater (Quorum Q150T ES, Quorum Technologies, Laughton, UK) equipped with a silver target (70-AG5710 Silver Target 99.97% pure, Micro to Nano V.O.F., Haarlem, Netherlands) were used to prepare a set of samples with different AgNPs loadings, which depends on the sputtering time in the range of 10–60 s at 50 mA. The samples will be hereinafter referred to as *x* target_AgNPs/TNT, where x stands for the time of sputtering.

Table 1 presents the summary of the naming of the performed electrodes.

### 2.5. Electrochemical Measurements

The open circuit potential (OCP) tests and electrochemical impedance spectroscopy (EIS) scans were recorded using a standard three-electrode configuration with TNT or AgNPs/TNT platforms as the working electrode, standard silver chloride electrode (*E*_Ag/AgCl_ = 0.222 V vs. SHE) by Metrohm as the reference electrode, and the platinum mesh as auxiliary electrode. 

OCP measurements were carried out at room temperature (25 ± 2 °C) for 1800 s. EIS spectra were performed over a frequency range of 10^5^–0.1 Hz with a signal amplitude of 10 mV. All experiments were performed in PBS solution (0.01 M, 20 mL, pH 7.4).

All measurements were repeated three times (for three samples *n* = 3) using the potentiostat/galvanostat model PGSTAT 302N from Autolab (Metrohm).

## 3. Results and Discussion

### 3.1. Characterization of Reference Platforms: TNT and Ag/TNT

**Figure 1:**
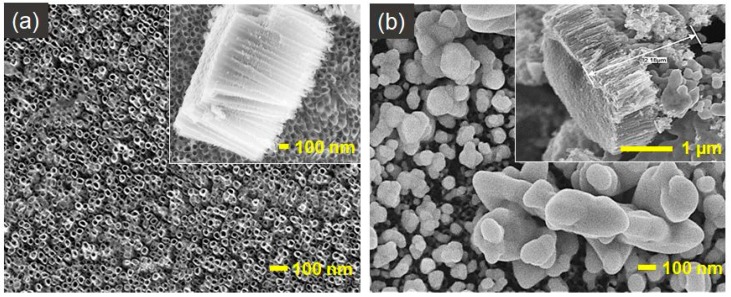
SEM top-view and cross-sectional (on insets) images of: (**a**) TNT and (**b**) Ag/TNT reference electrodes.

SEM images of TNT (Figure 1a) show opened from the top, closed at the bottom, and vertically oriented regular nanotubes with 50 ± 5 nm outer diameter and 1000 ± 100 nm in length completely covering the titanium foil. No damage on the TNT layers after annealing at 450 °C for 2 h was observed. Figure 1b presents an irregular silver layer with an uneven height in which silver completely closes the nanotubular morphology of TNT (63.18 ± 8.60 % wt. of silver).

As a result of thermal treatment, the amorphous structure of the TiO_2_ (originally present in nanotube structures) changes into a crystalline form of rutile and/or anatase. The most important advantage of annealing is that it causes the formation of oxygen vacancies, which results in improved TNT conductivity, and thus facilitates the transfer of charge that is attributed to the conversion of Ti^4+^ to Ti^3+^ [37,38,39,40]. The crystalline phases indicate also more corrosion resistance than the amorphous phase [38]. Studies show that annealing in argon generates more oxygen vacancies in TiO_2_ than annealing in the air, oxygen, or nitrogen [41,42,43]. The TiO_2_ nanotubes annealed at 450 °C are characterized by the predominance of anatase in their structure [37,42,44], which is advantageous when using this substrate for biosensing, because anatase has a higher affinity for biomolecules than rutile [45]. On the other hand, Liang et al. [46] indicate that rutile structure may retard the formation of silver nanoparticles.

### 3.2. Characterisation of AgNPs/TNT Platforms with Silver Nanoparticles Obtained by Cyclic Voltammetry

**Figure 2:**
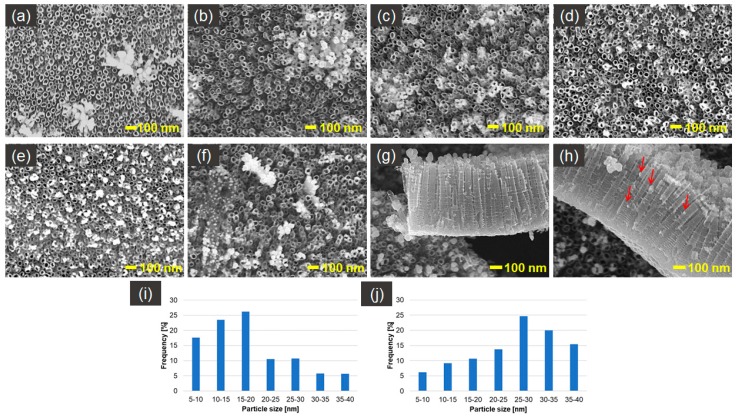
*SEM* top-view images of AgNPs/TNT platforms prepared by voltammetric method by: (**a**) 5, (**b**) 10, (**c**) 15, (**d**) 20, (**e**) 25, (**f**) 30 cycles, (**g**), (**h**) crss-sectional images of 25 cycles_AgNPs/TNT and histograms of particle-size distribution for: (**i**) 20 cycles_AgNPs/TNT, (**j**) 25 cycles_AgNPs/TNT.

Figure 2 presents SEM photographs of silver spherical and near-spherical nanoparticles on TNT platforms produced by cyclic voltammetry method, with the variable number of cycles. Only platforms after 20 and 25 cycles of CV of deposition do not contain silver nanoparticles agglomerates, and their size is between 5–40 nm. Distribution of the AgNPs after 20–25 cycles of CV (Figure 2d,e) of deposition is regular and well organized. Nanoparticles are concentrated mostly around nanotube edges, which can be caused by higher electric current density in those places [47]. The AgNPs, to a lesser extent, also fill the space between the nanotubes. Moreover, cross-sectional images of AgNPs/TNT (Figure 2g,h) show that some nanoparticles permeate into the nanotubes and embed in their inner walls. The mechanism of AgNPs formation consists of the nucleation of Ag metal at the initial stage. Ag metal has not completely evolved, with masses of the agglomerates on the top of the tubes. Afterwards, the Ag nuclei are created, and they become nucleation and growth sites for silver nanoparticles. After a time, nanoparticles can connect with each other and form into agglomerates [46]. For that reason samples 5–15 cycles_AgNPs/TNT (Figure 2b–d) contain agglomerates, which after 20–25 cycles of CV separate, resulting in the creation of homogeneously dispersed nanoparticles on TiO_2_ nanotubes substrate. The layer becomes supersaturated and agglomerates are created again with an increasing number of cycles of CV (around 30 cycles).

The particle size distribution histograms for the 20 and 25 cycles_AgNPs/TNT samples determined from the SEM images are shown in Figure 2i,j, respectively. From Figure 2i, it is clear that the frequency peak for 20 cycles_AgNPs/TNT electrode comes at approximately 15 nm–25 nm, and particles, whose sizes range from 5 nm to 30 nm, account for about 88% of the total particles observed. For 25 cycles_AgNPs/TNT sample the frequency peak comes at 25–30 nm, and particles, whose sizes range from 20 nm to 40 nm, account for about 75%.

Table 2 presents the results of measurements of the stationary potential of AgNPs/TNT platforms produced by cyclic voltammetry method and sizes of the obtained nanoparticles. Platforms that contain AgNPs and Ag/TNT are characterized by higher OCP value in comparison to TNT (−66 ± 14 mV). Electrodes 10–15 cycles_AgNPs/TNT and Ag/TNT structure are characterized by positive OCP value. It results from the occurrence of a higher number of unreduced silver ions in the structure rich in agglomerates.

**Figure 3:**
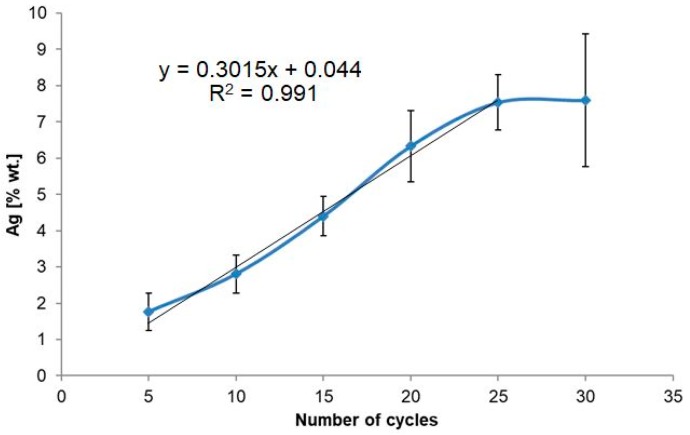
Chart presenting a number of deposition cycles dependence on silver weight content in produced AgNPs/TNT platforms.

Figure 3 shows that along with the increased number of cycles (up to 25) comes a linear growth (*R*^2^ = 0.991) of silver content, whereas at a later stage the silver content does not increase, probably due to layer saturation. 

**Figure 4:**
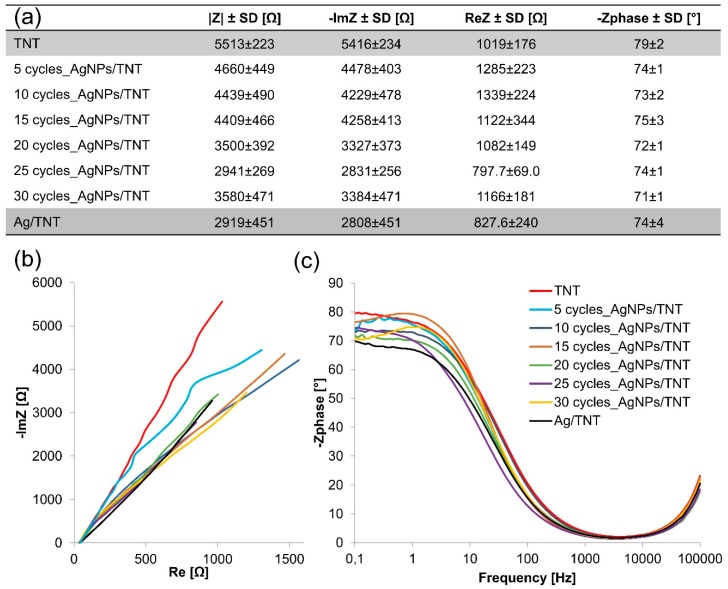
Summary of impedance parameters (**a**), Nyquist (**b**) and Bode (**c**) plots of AgNPs/TNT platforms differing in the number of cycles of silver nanoparticles deposition and reference layers: TNT and Ag/TNT. Spectra were recorded in the PBS solution (0.01 M, 10 ml, pH 7.4) over the frequency range 0.1–10^5^ Hz with amplitude 10 mV.

Figure 4a shows the Nyquist diagrams determined for AgNPs/TNT platforms differing in the number of cycles of silver nanoparticles deposition using cyclic voltammetry and for reference layers: TNT and Ag/TNT. The highest resistive character, which indicates the lower electrical conductivity, was observed for the TNT layer. On the other hand, the highest conductivity values were observed for AgNPs/TNT platform after 25 cycles of CV and for Ag/TNT (2919 ± 451 Ω). Both substrates are characterized by a similar value of impedance module, but the AgNPs/TNT platform (25 cycles) contains approximately 56% of silver weight less than Ag/TNT, which indicates that the elaborated electrode has a large surface area. Achieved results, according to results described in literature [48,49,50,51,52,53,54,55] in which the addition of silver nanoparticles is deposited onto various substrates, caused a decrease in the impedance module of the created sensor, in some cases even more than by 50%. Structures formed after 10, 15, and 30 cycles of deposition were characterized by lower conductivity, possibly due to the presence of agglomerates, which impede the electron transfer along a tubular structure. The phase angle values presented in Bode plots (Figure 4b) recorded in the lowest frequencies (0.1 Hz) in the range 79° to 71° are related to the porosity of the sample surface. The lowest porosity value of the phase angle was observed for the Ag/TNT. 

### 3.3. Characterization of AgNPs/TNT Platforms in which Silver Nanoparticles Were Obtained by Chronoamperometry

**Figure 5:**
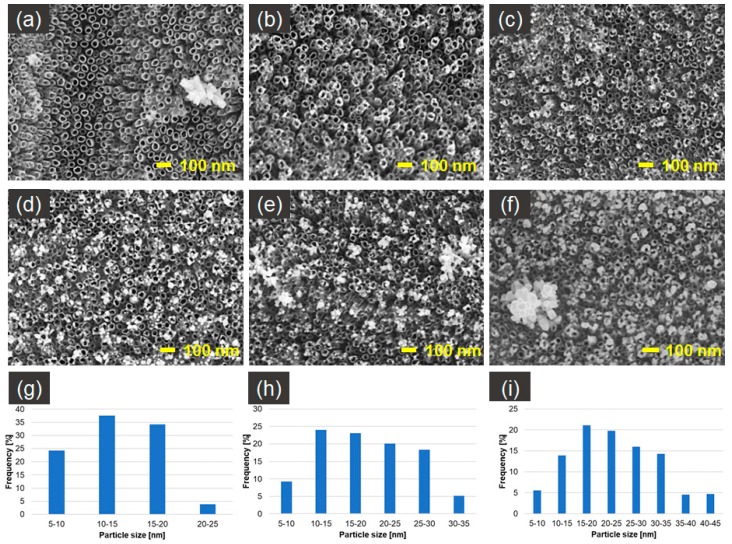
SEM top-view images of AgNPs/TNT platforms prepared by chronoamperometry for: (**a**) 30 s, (**b**) 60 s, (**c**) 120 s, (**d**) 180 s, (**e**) 240 s, (**f**) 300 s and histograms of particle-size distribution for: (**g**) 60 chrono_ AgNPs/TNT, (**h**) 120 chrono_ AgNPs/TNT, (**i**) 180 chrono_ AgNPs/TNT.

Figure 5 presents SEM photographs of silver spherical and near-spherical nanoparticles on TNT platforms produced by the chronoamperometry method where the duration of the process was a variable. Only platforms 60–180 chrono_AgNPs/TNT do not contain agglomerates and the distribution of AgNPs is regular and almost uniform, and the majority of them are present in the upper part of nanotubes, similar to the platforms produced by the CV method. The mechanism of nanoparticles formation corresponds to the one described in the previous subchapter.

The particle size distribution histograms for the 60–180_AgNPs/TNT samples determined from the SEM images are shown in Figure 5g–i. From Figure 5g, it is clear that the frequency peak for 60 and 120 chrono_AgNPs/TNT electrodes came at 10 nm–15 nm (Figure 5h) for 180 chrono_AgNPs/TNT at 15–20 nm (Figure 5i). For 25 cycles_AgNPs/TNT sample the frequency peak comes at 25–30 nm. As the deposition time increased, the spread of the obtained nanoparticles sizes increased.

**Figure 6:**
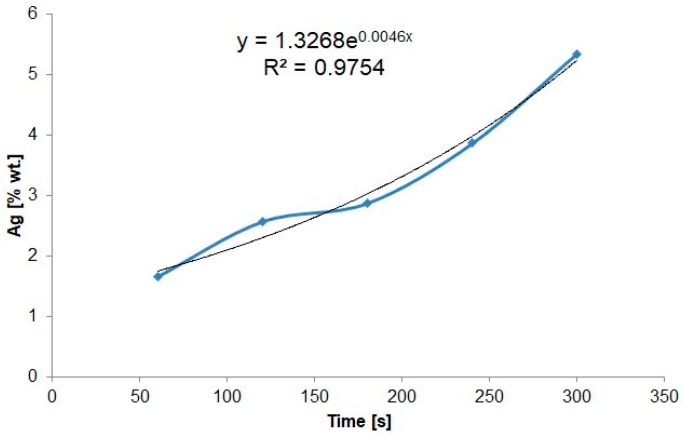
Chart of deposition duration dependency on the silver weight content in produced AgNPs/TNT platforms.

Table 3 presents the results of measurements of the stationary potential of AgNPs/TNT platforms produced by the chronoamperometry method and sizes of the obtained nanoparticles. Platforms that contain Ag are characterized by higher OCP value in comparison to TNT without nanoparticles. AgNPs/TNT obtained after 60 and 120 s of deposition are characterized by positive OCP value. A similar tendency has been observed in the voltammetric method of nanoparticles production. Initially, the nanoparticles were not stable and their surface area was very easily oxidized, but the longer deposition time indicated the greater nanoparticles’ stability, which results in lower OCP value (despite higher silver content in the structure in comparison to AgNPs/TNT produced after 60 and 120 s). Figure 6 shows that along with an increase in the process duration, there comes exponential growth of the silver content (*R*^2^ = 0.9754) in the obtained substrates.

**Figure 7:**
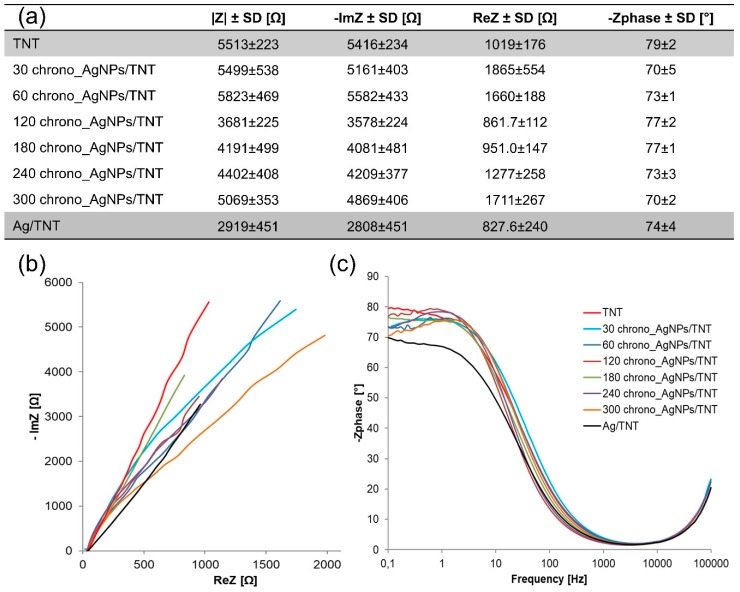
Summary of impedance parameters (**a**), Nyquist (**b**) and Bode (**c**) plots of AgNPs/TNT platforms differing in time of silver nanoparticles deposition and reference layers: TNT and Ag/TNT. Spectra were recorded in the PBS solution (0.01 M, 10 ml, pH 7.4) over the frequency range 0.1–10^5^ Hz with amplitude 10 mV.

Figure 7 shows the Nyquist diagrams determined for AgNPs/TNT platforms differing in time of silver nanoparticles deposition and for reference layers TNT and Ag/TNT. The addition of nanoparticles caused a decrease in the impedance module and thus improvement in electrical conductivity of the created platforms, which was connected to a large effective area facilitating the electron transfer [56]. The formation of agglomerates and plugging the nanotubes by nanoparticles has a negative effect on the structure conductivity, which is confirmed by research [57] showing that the highest impedance module is characteristic of the structures where the tubular structure is not blocked. The lowest impedance module is characteristic of the 120 chrono_AgNPs/TNT platform.

### 3.4. Characterisation of AgNPs/TNT Platforms in which Silver Nanoparticles Were Obtained by Sputter Deposition

**Figure 8:**
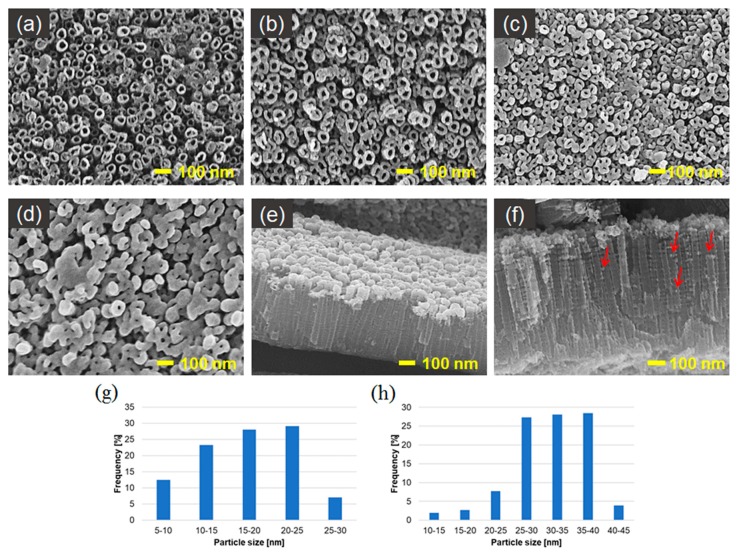
SEM top-view images of AgNPs/TNT platforms prepared by sputter deposition after: (**a**) 10 s, (**b**) 20 s, (**c**) 40 s, (**d**) 60 s, (**e**), (**f**) cross-sectional images of 20 target_AgNPs/TNT and histograms of particle-size distribution for: (**g**) 10 target__AgNPs/TNT, (**h**) 20 target_ AgNPs/TNT.

Figure 8 presents SEM micrographs of AgNPs/TNT platforms produced by sputter deposition. Figure 8a,b show that the distribution of AgNPs is regular, well organized and focused on the edges of nanotubes. The AgNPs, to a lesser extent, fill the space between the nanotubes and also permeate into the nanotubes and embed in their inner walls (Figure 8e,f). The nanoparticles have a spherical and near-spherical shape, and after 20 seconds of sputtering, AgNPs are located around the ring of nanotubes and the formation of nanoclusters was observed. The amount of silver was so large that it resulted in a visible reduction of their diameter. Over time, this layer increased in thickness, completely closing the tubular morphology. These studies are in accordance with the results obtained by Roguska et al [36].

The particle size distribution histograms for the 10 and 20 target_AgNPs/TNT samples determined from the SEM images are shown in Figure 8g,h, respectively. The frequency peak for 10 target_AgNPs/TNT electrode comes at 20 nm–25 nm, and particles, whose sizes range from 10 nm to 25 nm, account for about 80% of the total particles observed. For the 20 target_AgNPs/TNT sample the frequency peak came at approximately 30–40 nm.

**Figure 9:**
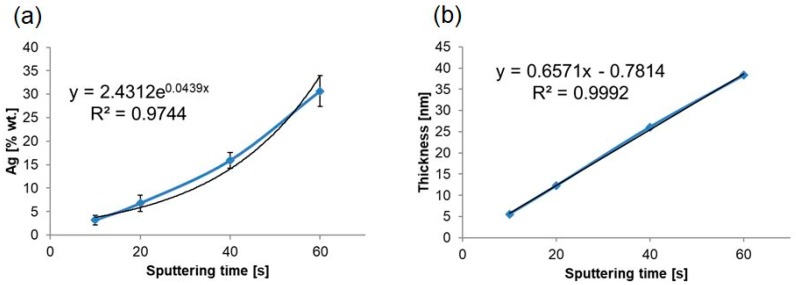
Chart presenting deposition duration dependency on the: (**a**) silver weight content and (**b**) thickness of silver layer in produced AgNPs/TNT platforms.

Figure 9 shows that along with the increase in process duration, there came exponential growth of the silver content (*R*^2^ = 0.9744) and a linear growth (*R*^2^ = 0.9992) of thickness of the silver layer in the obtained substrates.

**Figure 10:**
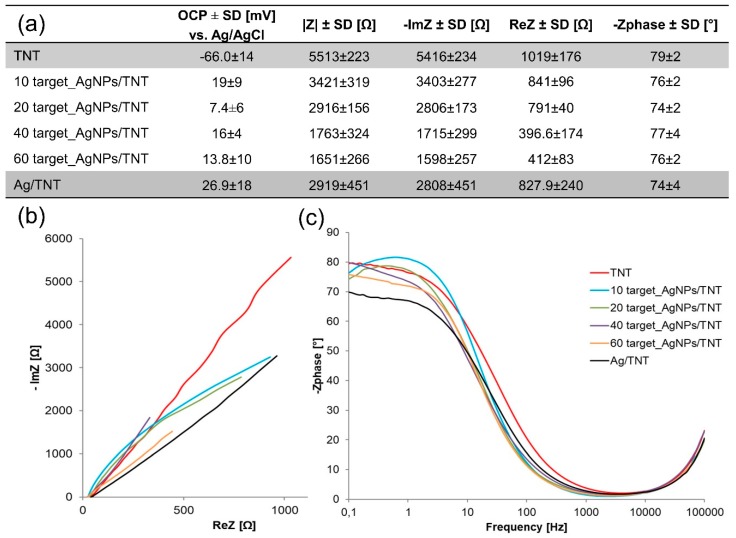
Summary of impedance parameters (**a**), Nyquist (**b**) and Bode (**c**) plots of AgNPs/TNT platforms differing in time of sputter deposition of silver and reference layers: TNT and Ag/TNT. Spectra were recorded in the PBS solution (0.01 M, 10 ml, pH 7.4) over the frequency range 0.1–10^5^ Hz with amplitude 10 mV.

The table in Figure 10 shows that adding silver to the structure caused an increase in the OCP values of created platforms. Figure 10a shows the Nyquist diagrams determined for AgNPs/TNT platforms differing in time of sputter deposition and for reference layers TNT and Ag/TNT. When the immersion time, and at the same time the silver weight content in the structure were increased, the impedance module was lowered, and for a sample after 60 s of sputtering it reached the value two times smaller than for Ag/TNT, but 60 target_AgNPs/TNT platform contained approximately 50% of silver weight less than the reference sample. It indicates that maintaining an open tubular structure is necessary for the efficient transport of electrons, which affects the conductivity of the platform. However, importantly, samples 40–60 target_AgNPs/TNT should be considered TNT platforms with silver nanoclusters, not with nanoparticles. The Bode plot (Figure 10b) shows that silver deposition reduces the phase angle of the AgNPs/TNT samples.

The greatest advantages of the sputter deposition and electrochemical production of nanoparticles are their ease and speed of implementation, as well as the possibility of controlling the process. Additionally applied methods do not require the stabilizing and reducing reagents. 

Each of the methods of AgNPs formation allowed to produce platforms whose impedance module was similar to reference substrate Ag/TNT. In the case of CV, it was 25 cycles_AgNPs/TNT sample, for CA – 120 chrono_AgNPs/TNT, and for SD – 20_target_AgNPs/TNT. 40–60 target_AgNPs/TNT are characterized by an impedance module almost 50% lower than Ag/TNT; however, these samples should be considered TNT platforms with silver nanoclusters, not with nanoparticles. Substrates containing silver agglomerates generated by electroreduction methods block the nanotubes by reducing the electrochemically active surface area and, therefore, also reduce the conductivity of AgNPs/TNT. In the case of sputtering, the nanoparticles agglomerate around the rings of the nanotubes without clogging them, and therefore do not reduce the conductivity of the electrodes; the greater the content of silver in the structure, the higher its conductivity.

In comparison to an impedance module equal to 5513 ± 223 Ω for TNT, in the case of electroreduction and sputter deposition methods, its value was reduced by approximately 50%. It is worth noting, however, that the aforementioned structures contained respectively 7.54 ± 0.76 % wt. Ag (25 cycles of CV), 2.57 ± 0.18 % wt. Ag (120 seconds of CA) and 6.79 ± 1.70 % wt. Ag (20 seconds of SD), and in the case of Ag/TNT (63.18 ± 8.60 % wt. Ag) it was 8–21 times higher content, which only confirms significant surface development of the created electrodes.

## 4. Conclusions

1. Produced silver spherical and near-spherical nanoparticles were concentrated mostly in the upper part of nanotubes around their rings and, to a lesser extent, also filled the space between the nanotubes and permeated into them. With the increase in duration of deposition, an increase in the spread of the size of nanoparticles, and an increase in the most frequent value of these sizes was observed. In the case of cyclic voltammetry, there occurred a linear growth of % silver weight content in the structure along with the increased number of cycles to 25, after which the layer became saturated. In the case of chronoamperometry and sputter deposition exponential growth of % wt. of Ag in time was noted.
2. Generally, the addition of silver to the structure increased the OCP value, which in turn increased the corrosion resistance of these structures. In the case of electrodeposition it can be noticed that a shorter duration of nanoparticles deposition/lower number of cycles results in creating platforms whose OCP has positive values. This probably occurs because of a greater number of unreduced silver ions in non-stabilized structures rich in agglomerates.
3. Nanoparticles caused a decrease in the impedance module (up to 50% lower when compared to TNT) and hence increased conductivity of the created electrodes. The highest conductivity among all samples without agglomerates was noted for the electrode after 25 cycles of AgNPs deposition by cyclic voltammetry (25 cycles_AgNPs/TNT).

## Figures and Tables

**Table 1:** Summary of the naming of the performed electrodes.

Sample	Description
***x* cycles_AgNPs/TNT**	TNT with AgNPs deposited using CV in 1 mM AgNO_3_; where *x* – number of cycles: *x* = 5, 10, 15, 20, 25, 30.
***x* chrono_AgNPs/TNT**	TNT with AgNPs deposited using CA in 1 mM AgNO_3_; where *x* – time of deposition: *x* = 30, 60, 120, 180, 240, 300.
***x* target_AgNPs/TNT**	TNT with AgNPs deposited using SD; where *x* – time of deposition: *x* = 10, 20, 40, 60.
**Ag/TNT**	TNT with silver micro layer deposited using CV in 50 mM AgNO_3_ for 25 cycles.

**Table 2:** The values of OCP and size of AgNPs deposited on TNT platforms prepared by voltammetric method.

	AgNPs/TNT						Ag/TNT
**Number of cycles**	5	10	15	20	25	30	25
**Size of AgNPs**	5–80 ^a^ nm	5–50 ^a^ nm	5–40 ^a^ nm	5–40 nm	5–40 nm	5–70 ^a^ nm	layer ^a^
**OCP [mV] versus Ag/AgCl**	−18.0 ± 24	8.11 ± 15	27.0 ± 23	−18.9 ± 9	−2.6 ± 2	−16.7 ± 13	26.9 ± 18

^a^ – platform with agglomerates.

**Table 3:** The values of OCP and size of AgNPs deposited on TNT platforms prepared by chronoamperometry method.

	AgNPs/TNT					
**Time [s]**	30	60	120	180	240	300
**Size of AgNPs**	5–30^a^ nm	5–25 nm	5–35 nm	5–45 nm	10–50 nm	10–70 ^a^ nm
**OCP [mV] versus Ag/AgCl**	4.00 ± 19	21.6 ± 23	17.2 ± 13	−18.9 ± 20	−13.6 ± 12	−26 ± 1

^a^ – platform with agglomerates.
